# A crucial role for the C‐terminal domain of exported protein 1 during the mosquito and hepatic stages of the *Plasmodium berghei* life cycle

**DOI:** 10.1111/cmi.13088

**Published:** 2019-07-30

**Authors:** Kamil Wolanin, Diana Fontinha, Margarida Sanches‐Vaz, Britta Nyboer, Kirsten Heiss, Ann‐Kristin Mueller, Miguel Prudêncio

**Affiliations:** ^1^ Parasitology Unit, Centre for Infectious Diseases Heidelberg University Hospital Heidelberg Germany; ^2^ Instituto de Medicina Molecular João Lobo Antunes, Faculdade de Medicina Universidade de Lisboa Lisboa Portugal; ^3^ German Centre for Infection Research, Heidelberg Division Heidelberg Germany; ^4^ PEPperPRINT GmbH, Research & Development Division Heidelberg Germany

**Keywords:** cell interaction, infection, membrane, microbial, protozoa

## Abstract

Intracellular *Plasmodium* parasites develop inside a parasitophorous vacuole (PV), a specialised compartment enclosed by a membrane (PVM) that contains proteins of both host and parasite origin. Although exported protein 1 (EXP1) is one of the earliest described parasitic PVM proteins, its function throughout the *Plasmodium* life cycle remains insufficiently understood. Here, we show that whereas the N‐terminus of *Plasmodium berghei* EXP1 (*Pb*EXP1) is essential for parasite survival in the blood, parasites lacking *Pb*EXP1's entire C‐terminal (CT) domain replicate normally in the blood but cause less severe pathology than their wild‐type counterparts. Moreover, truncation of *Pb*EXP1's CT domain not only impairs parasite development in the mosquito but also abrogates *Pb*EXP1 localization to the PVM of intrahepatic parasites, severely limiting their replication and preventing their egress into the blood. Our findings highlight the importance of EXP1 during the *Plasmodium* life cycle and identify this protein as a promising target for antiplasmodial intervention.

## 1 INTRODUCTION

Malaria is the most deadly parasitic disease worldwide, having claimed an estimated 435,000 lives in 2017, most of which in the poorest regions of the globe and of children under the age of 5 years old (World Health Organization, 2018). The disease is caused by *Plasmodium* parasites, eukaryotic organisms with a complex life cycle involving two obligatory hosts and multiple infection stages. Mammalians are infected when female *Anopheles* mosquitoes deposit sporozoites (spz) in their skin during a blood meal. Spz then travel in the bloodstream and eventually arrest in the liver sinusoids. After traversing several cells, spz productively infect a hepatocyte, initiating the asymptomatic but obligatory liver stage of infection. Intrahepatic parasites are enclosed within a parasitophorous vacuole (PV) membrane (PVM), inside which they differentiate into round‐shaped exoerythrocytic forms. A process of massive replication ensues, culminating in the formation of thousands of blood‐infective merozoites, which are eventually released into the blood stream, initiating the blood stage of infection (Prudencio, Rodriguez, & Mota, 2006). During this phase, merozoites cyclically invade, replicate inside, and burst red blood cells, leading to pathology. Concomitantly, a few blood stage parasites differentiate into male and female gametocytes, the parasite's sexual forms that can be ingested by an *Anopheles* mosquito during a subsequent blood meal. The sexual stage of the *Plasmodium* life cycle occurs inside the mosquito and is initiated by the differentiation of gametocytes into gametes, which fuse to generate zygotes. These forms then transform into ookinetes, which penetrate the mosquito's midgut (MG) wall and develop into oocysts. The latter undergo a process of maturation, during which spz are formed, eventually leading to oocyst rupture and sporozoite release. Free spz eventually invade the mosquito's salivary glands (SGs), where they remain ready to initiate a new mammalian infection (Meibalan & Marti, 2017).

*Plasmodium falciparum* (*Pf*) exported protein 1 (EXP1), previously known as antigen 5.1 (Hope, Hall, Simmons, Hyde, & Scaife, 1984), circumsporozoite‐related antigen (Coppel et al., 1985), or antigen QF116 (Kara et al., 1988), is a 17.1‐kDa PVM protein with a 162 amino acid (aa) long sequence, which is highly conserved among the *Plasmodium* species (Doolan et al., 1996). EXP1 is expressed by the parasite's blood stages (Hope et al., 1984; Simmons, Woollett, Bergin‐Cartwright, Kay, & Scaife, 1987) and is essential for parasite proliferation during this phase of infection (Maier et al., 2008). EXP1 has recently been shown to localize to dense granules in merozoites and to translocate to the PVM after erythrocyte invasion (Iriko et al., 2018), where it has been proposed to function as a glutathione S‐transferase (GST; Lisewski et al., 2014). EXP1 is also expressed by liver stage parasites (Sanchez, Rogers, Mellouk, & Hoffman, 1994) and localizes to the PVM, to which it is continuously transported throughout at least the first 30 hr of intrahepatic development (Hanson et al., 2013). We have recently shown that *Plasmodium berghei* (*Pb*) EXP1 binds to host Apolipoprotein H (ApoH) during the hepatic phase of infection and that this interaction plays a pivotal role throughout parasite development inside liver cells (Sa et al., 2017).

*Pb*/*Pf*EXP1's topology includes an N‐terminal (NT) domain (aa 1–75/1–79) that harbours a classical signal peptide (aa 1–23; Coppel et al., 1985); a putative catalytic domain that includes the *Pf* Arg70 residue and is presumed to be responsible for GST activity in blood stage parasites (Lisewski et al., 2014); an internal hydrophobic region typical of transmembrane anchor sequences (aa 75–97/79–101; Coppel et al., 1985); and a C‐terminal (CT) domain (aa 97–166/101–162) that includes a C1 (aa 103–136/107–134) and a C2 (aa 137–166/135–162) region, as defined in Sa et al. (2017). In both blood and liver parasite stages, EXP1 is proposed to be integrated into the PVM with the N‐terminus facing the lumen of the PV and the C‐terminus exposed to the host cell cytosol (Ansorge, Paprotka, Bhakdi, & Lingelbach, 1997; Gunther et al., 1991; Tribensky, Graf, Diehl, Fleck, & Przyborski, 2017). It has recently been reported that *Pf*EXP1 trafficking to the PVM happens independently of the putative *Plasmodium* translocon of exported proteins (PTEX; de de Koning‐Ward et al., 2009) and does not require unfolding of the protein (Tribensky et al., 2017).

We have previously shown that the C2 region of *Pb*EXP1's CT domain mediates the interaction with, and internalisation of, host ApoH during the hepatic stage of infection (Sa et al., 2017). However, little is known about the role of each domain of this protein, including the CT domain in its entirety, on the various stages of the parasite's life cycle. Here, we show that the NT domain of *Pb*EXP1 is essential for blood stage development of the parasite and is, therefore, refractory to deletion or manipulation. Conversely, parasites bearing a version of *Pb*EXP1 that lacks the entire CT domain were successfully generated and analysed throughout their life cycle. We show that this truncation does not affect parasite replication in the blood but severely impairs its development in the mosquito, as well as its ability to infect and replicate inside liver cells, abrogating EXP1 localization to the PVM and impairing parasite egress to the blood.

## 2 RESULTS

### 2.1 The CT domain of PbEXP1 is not essential for blood stage parasite development

In order to dissect the functional role of different domains of *Pb*EXP1, we attempted to generate modified versions of this protein bearing a truncated version of either its NT or its CT domain. Despite multiple attempts, we were unable to generate a modified version of *Pb*EXP1 lacking the NT domain (*Pb*EXP1ΔNT, Figure S1a,b), suggesting that it is essential for blood stage development of *Pb* parasites. However, transgenic *Pb* parasite lines expressing a CT domain‐truncated version of *Pb*EXP1 were successfully generated in both the *Pb*ANKA (*Pb*WT) and *Pb*GFPcon (*Pb*_GFP_) parental parasite lines, yielding their *Pb*EXP1ΔCT and *Pb*_GFP_EXP1ΔCT mutated counterparts, respectively (Figure S1c–h), and showing that *Pb* blood stages can develop in the absence of the *Pb*EXP1 CT domain. Successful deletion of the CT domain in the *Pb*EXP1ΔCT parasites was confirmed by Western blot using two different anti‐EXP1 antibodies that bind either to epitopes present on the full‐length *Pb*EXP1 protein (Figure S1e) or to epitopes present only on the protein's C‐terminus (Figure S1f). Our results further show that the onset and initial progression of parasitaemia following injection of *Pb*EXP1ΔCT‐infected red blood cells (iRBC) in C57BL/6 mice are indistinguishable from those observed in mice infected with a similar inoculum of *Pb*WT‐iRBC (Figure S2a,b). Interestingly, however, whereas five out of six mice infected with *Pb*WT‐iRBC succumbed to experimental cerebral malaria (ECM) within the expected timeframe (Zuzarte‐Luis, Mota, & Vigario, 2014), none of the *Pb*EXP1ΔCT‐iRBC‐infected mice developed ECM symptoms and died later with hyperparasitaemia (Figure S2c). Collectively, our results confirm the non‐essentiality of *Pb*EXP1's CT domain for parasite growth in the blood, while suggesting a possible role for *Pb*EXP1 on malaria pathology and disease severity.

### 2.2 Truncation of the CT domain of PbEXP1 impairs parasite sporogony and intrahepatic development in vitro and in vivo

**Figure 1:**
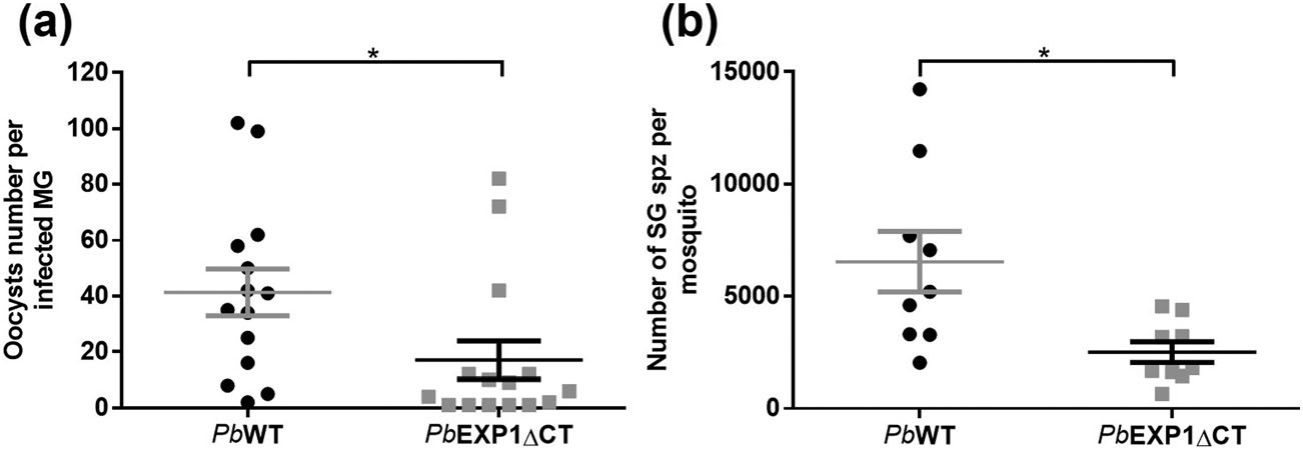
Impact of CT truncation on the sporogony of the PbEXP1ΔCT parasite. (a) Total number of oocysts per infected midgut (MG). MG counts were performed in mosquitoes from three independent infections with the PbWT (n = 14) and PbEXP1ΔCT parasites (n = 15). (b) Quantification of the number of sporozoites (spz) present in salivary glands (SGs) of PbWT‐ and PbEXP1ΔCT‐infected mosquitoes. The pooled data of mean number of sporozoites per mosquito, per infection, from nine independent mosquito infections and 40–120 mosquitoes dissected per infection are shown. Panels (a) and (b) were analysed using two‐tailed unpaired t test. For panel (a), ^*^ P = .0321, and for panel (b), ^*^ P = .0123. Data are shown as mean ± SEM.

Having shown that *Pb* parasites expressing a CT‐truncated version of *Pb*EXP1 retain their ability to replicate in the blood, we proceeded to examine the effect of the CT deletion on the remaining stages of the parasite's life cycle. To this end, mosquitoes were allowed to feed on the blood of *Pb*EXP1ΔCT‐ or *Pb*_GFP_EXP1ΔCT‐infected mice, as well as on mice infected with each of these parasites' parental lines, employed as controls. The numbers of oocysts developing in the mosquitoes' MGs, and of spz present in the mosquitoes' SGs, were then determined 10–12 and 17–21 days after infection, respectively. Our results show that *Pb*EXP1ΔCT‐infected mosquitoes present significantly lower numbers of oocysts per MG than mosquitoes infected with *Pb*WT parasites (Figure 1a). As expected, this decrease is mirrored by that observed in the numbers of SG spz in *Pb*EXP1ΔCT‐ and *Pb*_GFP_EXP1ΔCT‐infected mosquitoes relative to those present in mosquitoes infected with the corresponding control parasites (Figures 1b and S3a). Of note, an estimation of the relative proportions of spz in the MG and in the SG indicates that the deletion of *Pb*EXP1's CT domain does not impair the parasite's ability to exit the oocysts and invade the SG (Figures S3b and S4). Overall, these results suggest that deletion of the CT domain of *Pb*EXP1 does not affect the migratory capacity of spz but severely impairs the parasite's ability to form oocysts in the mosquito MG.

**Figure 2:**
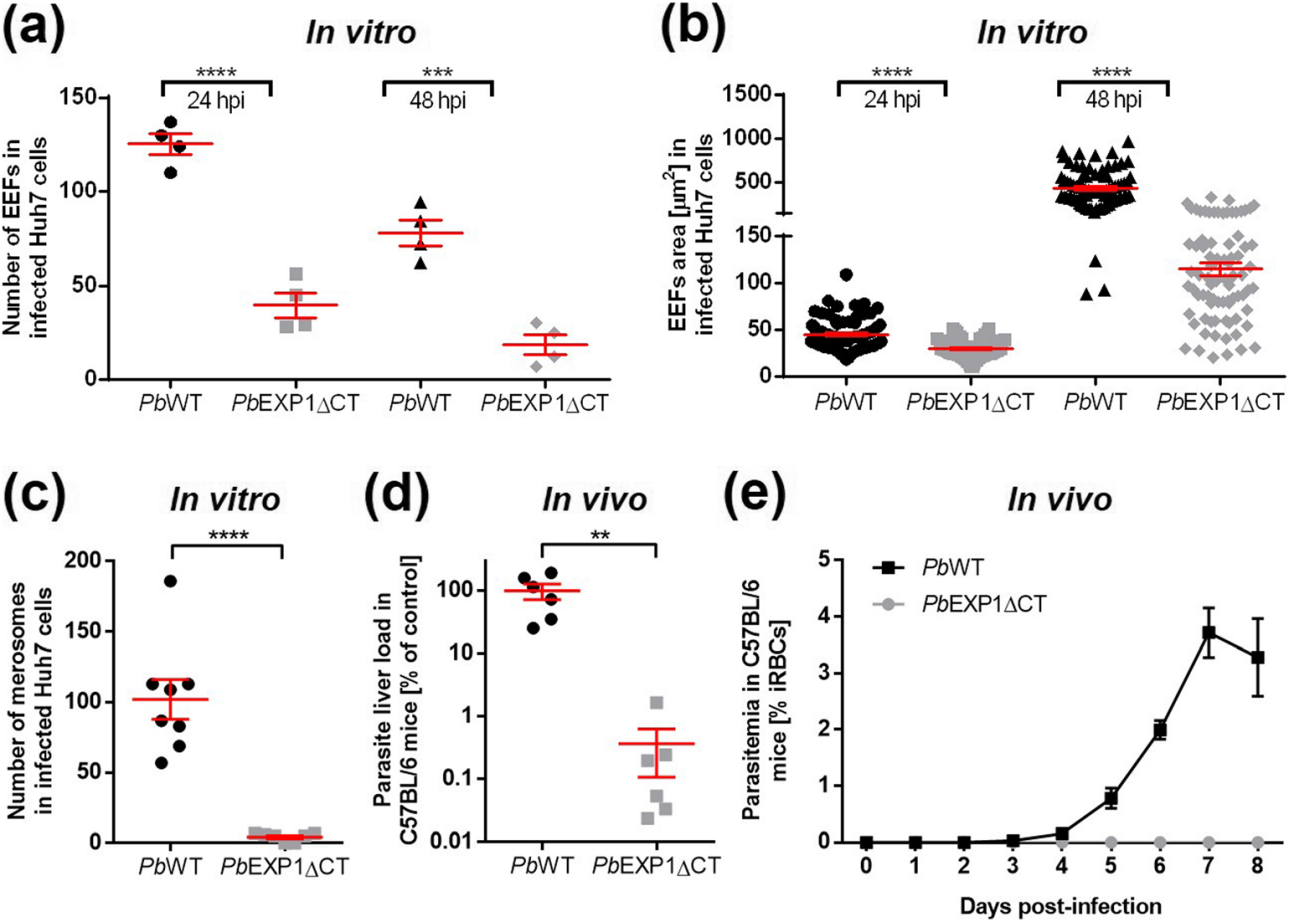
Impact of CT truncation on in vitro and in vivo hepatic infections by the PbEXP1ΔCT parasite. (a) Number of PbWT and PbEXP1ΔCT exoerythrocytic forms (EEFs) in infected Huh7 cells at 24 and 48 hr post‐infection (hpi) in four independent wells of eight‐well Lab‐Tek chamber slides. (b) Measurement of EEF size in infected Huh7 cells, using immunofluorescent microscopy, at 24 and 48 hpi. Results shown are from two independent experiments. Each symbol represents one parasite. n = 82 (for PbWT) and 79 (for PbEXP1ΔCT) for 24 hpi, and n = 81 (for PbWT) and 80 (for PbEXP1ΔCT) for 48 hpi. (c) Number of merosomes formed 72 h after infection of Huh7 cells with PbWT or PbEXP1ΔCT sporozoites. Results shown are from two independent experiments. Each symbol represents one well of an eight‐well Lab‐Tek chamber slide (n = 8). (d) Relative parasite burden in the livers of C57BL/6 mice infected by intravenous injection of 1 × 10^4^ PbWT or PbEXP1ΔCT sporozoites, measured by qRT‐PCR at 42 hpi. Parasite burden is represented as the level of transcription of parasite 18S rRNA normalised to the PbWT control, which was set to 100%. Results shown are from two independent experiments. Each symbol represents one mouse (n = 6). (e) PbWT and PbEXP1ΔCT parasitaemia curves following intravenous injection of 1 × 10^4^ sporozoites into C57BL/6 mice (n = 3). The percentage of infected red blood cells (iRBCs) was quantified by microscopic analysis of Giemsa‐stained blood smears. Panels (a) to (d) were analysed using the two‐tailed unpaired t test. For panel (a), ^****^ P < .0001 and ^***^ P = .0003; for panel (b), ^****^ P < .0001; for panel (c), ^****^ P < .0001; and for panel (d), ^**^ P = .0047. Data are shown as mean ± SEM.

We next sought to investigate the impact of the truncation of *Pb*EXP1's CT domain on *Pb*'s hepatic infectivity. We started by performing in vitro infections of Huh7 hepatoma cells with spz of both mutant parasites and their respective control lines and monitoring infection by immunofluorescence microscopy. Our results show that both the *Pb*EXP1ΔCT and *Pb*_GFP_EXP1ΔCT parasites display a significant reduction in the number of infected cells analysed 24 and 48 hr post‐infection (hpi; Figures 2a and S3c), as well as in the size of the exoerythrocytic forms replicating intrahepatically at these time points (Figures 2b and S3d), relative to their corresponding controls. Additionally, our data show that the number of *Pb*EXP1ΔCT merosomes formed at 72 hpi is markedly decreased compared with that observed for *Pb*WT control parasites (Figure 2c). These data suggest that truncation of the *Pb*EXP1 CT domain severely impairs the parasite's ability to establish a hepatic infection, as well as to replicate inside liver cells. To determine whether the impairment seen in vitro was also observed in an in vivo setting, mice were infected by injection of similar numbers of spz of each of the mutant and control parasite lines. Quantification of hepatic infection, at 42 hpi, showed that the parasite burden in the livers of *Pb*EXP1ΔCT‐ and *Pb*_GFP_EXP1ΔCT‐infected mice was drastically reduced compared with that of mice infected with the corresponding control parasite lines (Figures 2d and S3e). Importantly, when infection was allowed to proceed to the ensuing erythrocytic stage following sporozoite injection, parasites were detected in the blood of all *Pb*WT spz‐infected mice, whereas all the mice infected with *Pb*EXP1ΔCT spz remained blood stage negative (Figure 2e). Collectively, our data demonstrate that the CT domain of the *Pb*EXP1 protein plays a critical role on the parasite's ability to develop in liver cells and proceed to the subsequent blood stage of infection.

### 2.3 Truncation of the CT domain of PbEXP1 compromises the protein's localization and function during the liver stage of infection

**Figure 3:**
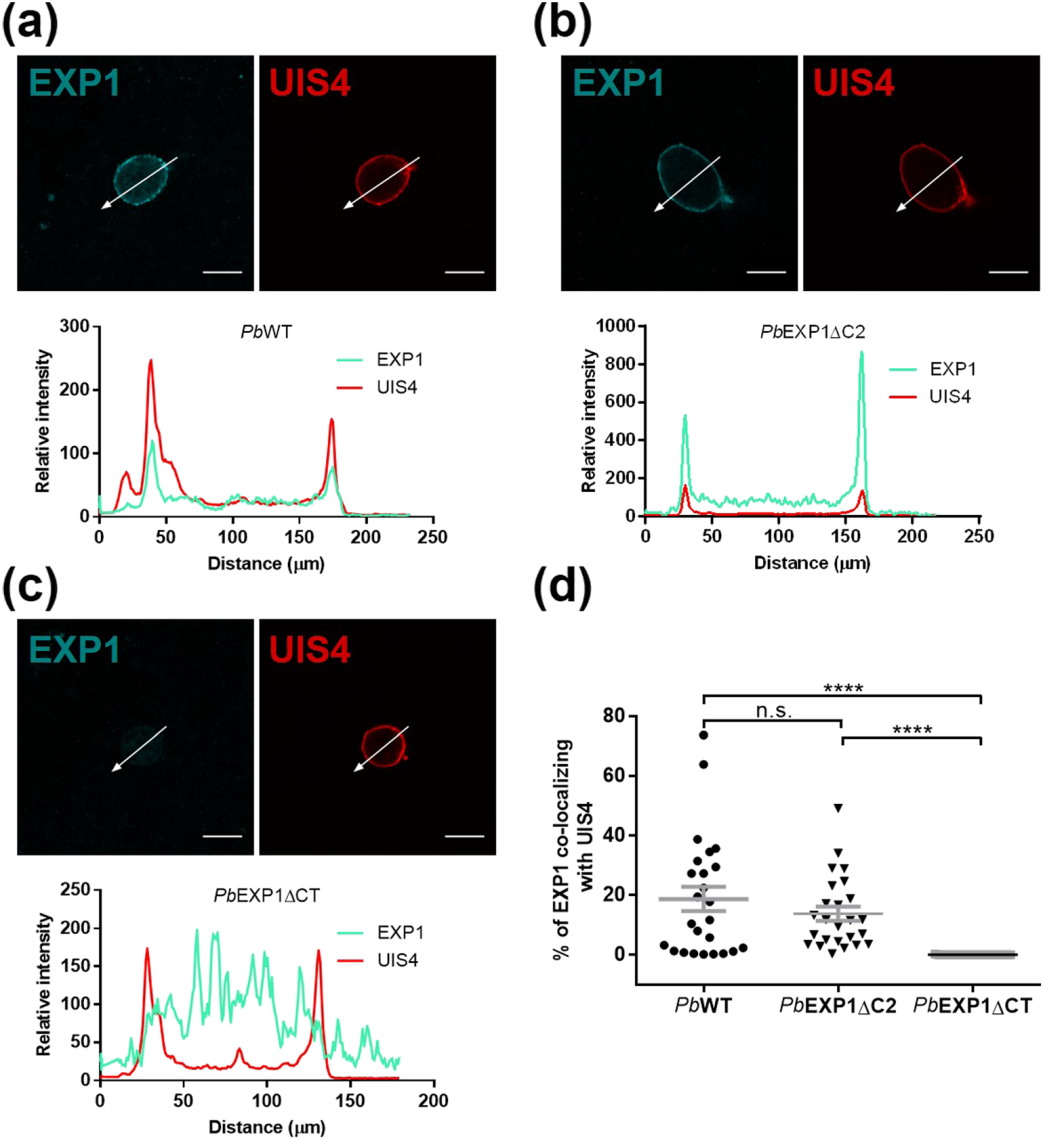
Impact of CT truncation on the PVM localization of PbEXP1. Colocalization of PbEXP1 (cyan) and UIS4 (red) proteins was assessed by immunofluorescence microscopy at 30 hpi, in Huh7 cells infected with 2 × 10^4^ sporozoites of (a) PbWT, (b) PbEXP1ΔC2, and (c) PbEXP1ΔCT parasite lines. (a–c) Upper panels: Representative immunofluorescence microscopy images (scale bar = 10 μm); lower panels: histograms showing the relative intensity of the cyan and red signals across the section indicated in the representative images by an arrow. (d) Quantification of EXP1/UIS4 colocalization, measured as the percentage of total EXP1 that colocalizes with UIS4. Results shown are from two coverslips infected in one experiment. Each symbol represents one parasite (n = 25). Data were analysed using two‐tailed unpaired t test. n.s., non‐significant. ^****^ P < .0001. Data are shown as mean ± SEM.

We have previously shown that the localization of *Pb*EXP1 on the PVM inside hepatic cells is not compromised in *Pb*EXP1ΔC2 parasites, which lack the C2 region of the protein's CT (Sa et al., 2017). To ascertain the impact of the truncation of the entire CT domain on *Pb*EXP1's ability to localize to the PVM, we employed immunofluorescence microscopy to analyse protein localization in Huh7 cells infected with *Pb*WT, *Pb*EXP1ΔC2, or *Pb*EXP1ΔCT parasites, using upregulated in spz (UIS) 4 as a marker of the PVM. Our results show that whereas, in agreement with our previous observations, *Pb*EXP1 colocalizes with UIS4 at the PVM in both *Pb*WT and *Pb*EXP1ΔC2 parasites (Figure 3a,b), *Pb*EXP1/UIS4 colocalization is completely abrogated in *Pb*EXP1ΔCT‐infected cells, with *Pb*EXP1 remaining contained inside the PV (Figure 3c,d). Not surprisingly, we also found that *Pb*EXP1ΔCT parasites fail to internalise ApoH, similarly to what was previously observed for the *Pb*EXP1ΔC2 mutant (Figure S5; Sa et al., 2017). Altogether, our results suggest that the CT domain of *Pb*EXP1 plays a pivotal role on the trafficking of the protein to the PVM, leading to its accumulation inside the hepatic PV.

## 3 DISCUSSION

The obligate intracellular hepatic and erythrocytic stages of *Plasmodium* parasites reside inside a PV, where they undergo a process of development and replication. The PVM that surrounds this compartment constitutes the de facto host cell–parasite interface during these phases of the parasite's life cycle and is regarded as a key player in several pivotal processes during infection, including protein export from the parasite to the host cell and nutrient transport to the parasite (Agop‐Nersesian, Niklaus, Wacker, & Theo Heussler, 2018; Tribensky et al., 2017). The PVM contains proteins derived both from the host (Bietz, Montilla, Kulzer, Przyborski, & Lingelbach, 2009; Posfai et al., 2018) and from the parasite (Spielmann, Gardiner, Beck, Trenholme, & Kemp, 2006; Spielmann, Montagna, Hecht, & Matuschewski, 2012). Among the latter are the five known components of the PTEX translocon and the EXP1 protein. Interestingly, both of these appear to play a different role during either the hepatic or the erythrocytic stages of *Plasmodium* development. In fact, although PTEX is a bona fide translocon essential for transport of effector proteins into host erythrocytes (Ho et al., 2018), it has been suggested that it may play a different role during the liver stage of infection, possibly taking up small molecules from the hepatocyte or contributing to the insertion of parasite proteins into the PVM (Kalanon et al., 2016). Likewise, although EXP1 was suggested to be a GST that conjugates glutathione onto haematin and plays a potential role in the mode of action of chloroquine during the blood stage of infection (Lisewski et al., 2014; Lisewski et al., 2018), we have recently shown that it interacts with host hepatic ApoH and likely mediates its internalisation during the liver stage of the *Pb* life cycle (Sa et al., 2017).

Despite being one of the earliest described parasite‐derived PVM proteins (Coppel et al., 1985; Kara et al., 1988; Simmons et al., 1987), much remains to be understood about EXP1. In the present study, we sought to dissect the role of the protein's NT and CT domains during the life cycle of the malaria parasite. It has previously been shown that EXP1 is refractory to deletion from both the *Pf* (Maier et al., 2008) and *Pb* (Sa et al., 2017) genomes, indicating its essentiality during blood stage parasite growth, where gene targeting is performed. We now show that deletion of the NT domain of *Pb*EXP1 also renders the parasite inviable, showing that this protein domain is essential during blood stage development. Conversely, we were able to generate *Pb* parasites expressing a truncated version of EXP1 that lacks the entire CT domain. However, the development of the *Pb*EXP1ΔCT parasite's mosquito stages is severely compromised, showing that, although asexual blood stage parasites can survive in the absence of *Pb*EXP1's CT domain, its deletion affects the parasite's development in the mosquito vector. Interestingly, it has been shown that *Plasmodium* parasites upregulate the mosquito precursor of Apolipophorin II/I, a key circulating lipid transport regulator whose silencing markedly decreases both *Pb* (Vlachou, Schlegelmilch, Christophides, & Kafatos, 2005) and *Pf* (Mendes et al., 2008) oocyst numbers. In combination with our own findings, this suggests that, similarly to its role during the liver stage of the *Plasmodium* life cycle, EXP1 may interact with host apolipoproteins during the parasite's development in the mosquito.

**Figure 4:**
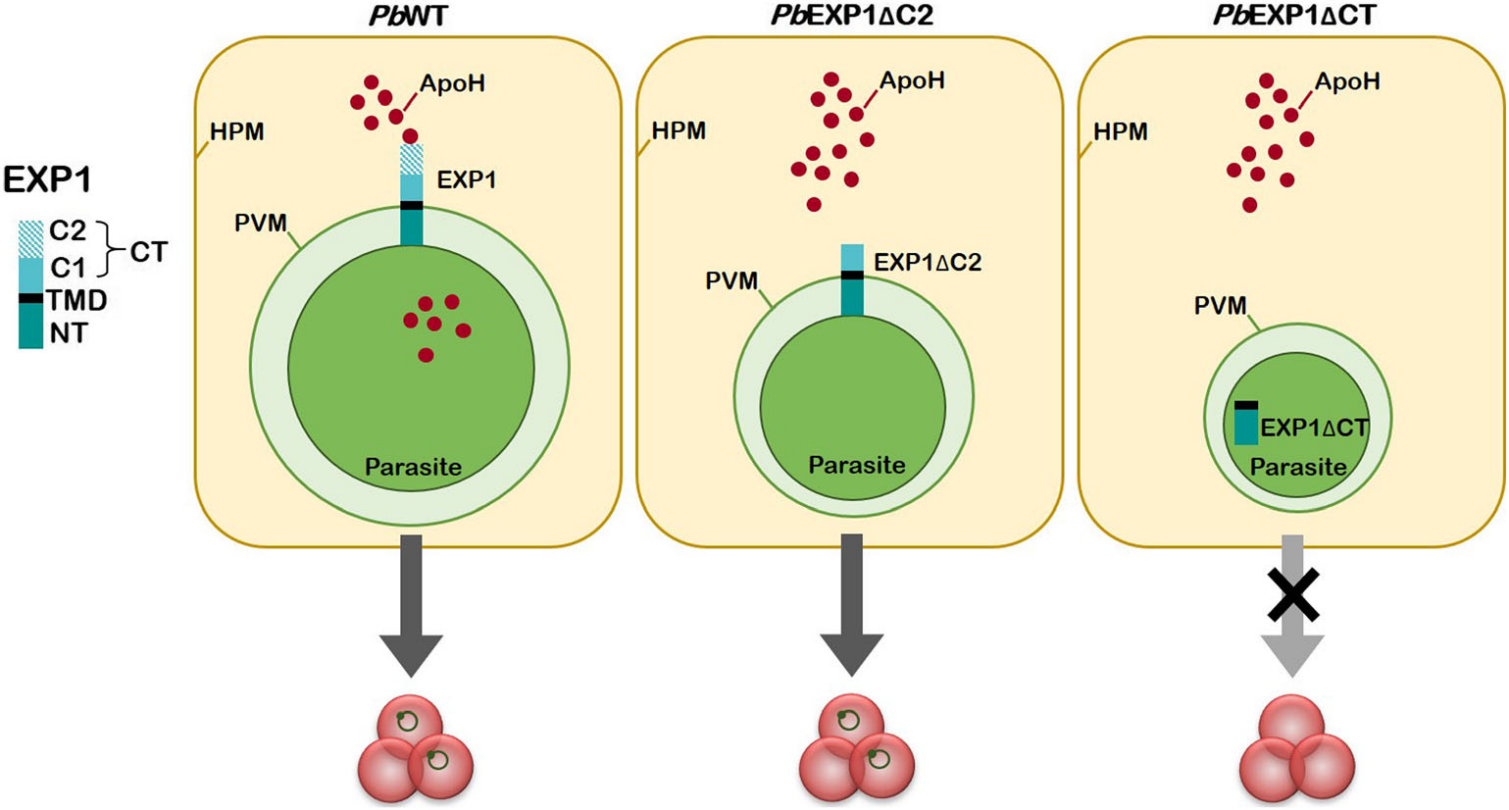
Schematic representation of EXP1's localization and function during the liver stage of infection by Plasmodium berghei. The C2 region of EXP1 interacts with host Apoliprotein H (ApoH), enabling its internalisation and allowing for successful parasite replication that culminates on egress from the liver and subsequent infection of red blood cells. In the absence of the C2 terminal region of EXP1 (PbEXP1ΔC2 parasites; Sa et al., 2017), EXP1 still localizes to the PVM but no longer interacts with and internalises ApoH, resulting in impaired parasite replication but not preventing the appearance of the blood stage of infection. In the absence of the entire CT domain (PbEXP1ΔCT parasites), EXP1 no longer localizes to the PVM, resulting in the developmental arrest of the parasite and its inability to proceed to the blood stage of infection. CT, C‐terminus, including the C1 and C2 regions; HPM, hepatocyte plasma membrane; NT, N‐terminus; PVM, parasitophorous vacuole membrane; TMD, transmembrane domain.

*Pb*EXP1ΔCT parasites are unable to internalise ApoH and display an impairment in hepatic development. These phenotypes are similar to those observed for the *Pb*EXP1ΔC2 parasite and can therefore be ascribed to the C2 region of the EXP1 protein (Sa et al., 2017). Although the liver stages of *Pb* parasites lacking the C2 region of EXP1 are no longer able to uptake host ApoH, *Pb*EXP1 retains its ability to localize to the PVM (Sa et al., 2017). We now show that PVM targeting of EXP1 during *Pb* hepatic development is completely abolished in the absence of the protein's entire CT domain, indicating that EXP1 localization is dictated by the C1 region of the protein. Importantly, these results are validated by the demonstration that a complementation parasite line (*Pb*EXP‐1compl) displays hepatic infection and EXP1 localization/function similar to those of its wild‐type counterpart (Sa et al., 2017). Thus, although a topology where EXP1's NT domain is exposed to the host cell cytosol and the CT domain faces the parasite PV lumen has been proposed (Lisewski et al., 2014), our results favour the widely preferred view that the CT protrudes into the host cell cytosol, whereas the NT faces the inside of the PV (Gunther et al., 1991; Tribensky et al., 2017). These results further suggest that the C1 and C2 regions of EXP1 play complementary roles in the protein's function, the former being responsible for protein targeting to the PVM, and the latter being involved in the uptake of host apolipoproteins (Figure 4).

Our work sheds light on the role of EXP1's NT and CT domains during the life cycle of *Plasmodium* parasites. We demonstrated the essentiality of the protein's NT domain for *Pb* growth in the blood, of its C1 region for PVM localization during liver infection, and of the entire CT domain for parasite development, both in the mosquito and in hepatic cells. Our findings lend support to the topology of EXP1 favoured by most of the available literature, according to which the protein's CT faces the host cell's cytosol, and suggest that this can serve as a target of novel antiplasmodial intervention strategies.

## 4 EXPERIMENTAL PROCEDURES

### 4.1 Ethics statement

All animal experiments were performed according to European regulations concerning the Federation for Laboratory Animal Science Associations and Society of Laboratory Animal Science standard guidelines. Animal experiments were approved by German authorities (Regierungspräsidium Karlsruhe), § 8 Abs. 1 Tierschutzgesetz (TierSchG) under license G260/12. For all experiments, 8‐week‐old female outbred NMRI mice were purchased from Charles River or Janvier Labs, and 8‐week‐old female inbred C57BL/6 mice were purchased from Janvier Labs. All mice were kept under specific pathogen‐free conditions within the animal facility of the University of Heidelberg (Interfakultäre Biomedizinische Forschungseinrichtung).

### 4.2 Cell culture

The Huh7 human hepatoma cell line was cultured in DMEM supplemented with 10% (v/v) FCS and 1% (v/v) antibiotic–antimycotic (100×) from Life Technologies. Cells were passaged by trypsinisation at ~100% confluence and maintained at 37°C with 5% CO_2_.

### 4.3 Mosquito rearing and parasite production

*Anopheles stephensi* mosquitoes were routinely reared at 28°C and 80% humidity and fed on a 10% (w/v) sucrose solution with 0.2 μg/ml of para‐aminobenzoic acid. Mice were infected by intraperitoneal injection of *Pb*WT or mutant parasites (cryopreserved iRBCs). Adult mosquitoes were starved and allowed to feed on infected mice and were subsequently maintained at 21°C and 80% humidity for up to 21 days. *Pb*ANKA (Hall et al., 2005) and *Pb*GFP_CON_ (*Pb*_GFP_; Janse et al., 2006) WT parasites, as well as transgenic *Pb*EXP1ΔC2 (Sa et al., 2017), *Pb*EXP1ΔCT, *Pb*EXP1ΔNT, and *Pb*_GFP_EXP1ΔCT spz, were obtained by dissection of SGs of infected female *A. stephensi* mosquitoes. Salivary gland suspensions were macerated and filtered through a 70‐μm cell strainer to remove mosquito debris. Free spz were then counted in a hemocytometer.

### 4.4 Generation of transgenic parasite lines

To generate the *Pb*EXP1ΔCT and *Pb*_GFP_EXP1ΔCT parasite lines, a 3′UTR fragment was amplified by using 3′UTR *Pb*EXP1for and 3′UTR *Pb*EXP1rev primers (Table S1), and a 5′ fragment including the ORF without the last 207 bp of *Pb*EXP1 was amplified with 5′UTR *Pb*EXP1 ORF dC2for (Sa et al., 2017) and 5′UTR *Pb*EXP1 ORF dCTrev primers (Table S1) from *Pb*WT genomic DNA (gDNA) and cloned into the b3D^+^ vector (Silvie, Goetz, & Matuschewski, 2008). A similar strategy was used to generate the *Pb*EXP1ΔNT parasite line. Briefly, a 5′ fragment without the last 114 bp coding NT domain of EXP1 protein after signal peptide (SP) was amplified using primers 5′UTR SP *Pb*EXP1for and 5′UTR SP *Pb*EXP1rev (Table S1) together with full transmembrane (TM) and CT domains, using primers TM CT *Pb*EXP1for and TM CT *Pb*EXP1rev (Table S1) from *Pb*WT gDNA, and cloned into the b3D^+^ vector containing the 3′UTR fragment, as described above. Before transfection, the targeting vectors were linearised by using restriction enzymes *KpnI* and *NotI*. To obtain clonal parasite populations, limited serial parasite dilutions were performed, and one parasite was administered by intravenous injection to each of 10 recipient naive NMRI mice (Thathy & Menard, 2002). After gDNA extraction, *Pb*WT, *Pb*EXP1ΔCT, *Pb*_GFP_EXP1ΔCT, and *Pb*EXP1ΔNT parasites were genotyped by using specific primers (Table S1). The truncations or mutations of each parasite line were additionally confirmed by sequencing.

### 4.5 Western blot

For Western blot analyses, blood samples were collected from *Pb*WT‐ and *Pb*EXP1ΔCT‐infected NMRI mice. Collected blood was diluted in PBS and centrifuged at 1,500 rpm for 8 min at room temperature (RT). The supernatant was removed, and the remaining erythrocyte pellet was lysed in 0.2% (v/v) saponin in PBS, followed by centrifugation at 2,800 rpm for 8 min at RT. The supernatant was discarded, and the pelleted parasites were resuspended in PBS and centrifuged at 7,000 rpm for 2 min at RT. Following removal of the supernatant, parasites were resuspended in RIPA buffer and incubated at −20°C overnight. Protein lysates were then mixed with Laemmli sample buffer 1:1, heat denatured at 95°C for 5 min, separated by SDS‐PAGE on BisTris 4–12% gradient gels, and transferred to methanol‐activated PVDF membranes. Membranes were blocked in 5% (w/v) of powdered milk in PBS containing 0.05% (v/v) Tween 20 (PBST) for 1 h at RT. Chicken anti‐*Pb*EXP1 FL (1:700; kindly provided by Prof. Heussler, University of Bern, Switzerland), mouse anti‐*Pb*HSP70 (1:200; Tsuji, Mattei, Nussenzweig, Eichinger, & Zavala, 1994), and rat anti‐*Pb*EXP1 C‐terminus (1:100; kindly provided by Dr. Julia Sattler, University of Heidelberg, Germany) primary antibodies were then diluted in blocking solution and incubated overnight at 4°C. Following three washes with PBST, membranes were incubated with the appropriate horseradish peroxidase‐conjugated secondary antibodies, diluted in blocking solution, for 1 hr at RT. Secondary antibodies used were donkey anti‐chicken IgG‐Peroxidase (1:5,000; Jackson ImmunoResearch, PA, USA), goat anti‐mouse IgG‐Peroxidase (1:10,000; Sigma‐Aldrich, MO, USA), and goat anti‐rat IgG‐Peroxidase (1:5,000; Jackson ImmunoResearch, PA, USA). Membranes were then washed three times with PBST, and the blots were developed by the addition of a chemiluminescent substrate (Western Lightning Plus‐ECL, PerkinElmer). Images were acquired with the LiCor scanner and analysed using Image Studio Lite software.

### 4.6 Assessment of Plasmodium sporogonic stages

For assessment of *Plasmodium* sporogonic stages, female *A. stephensi* mosquitoes were infected with *Pb*WT and *Pb*EXP1ΔCT parasites as described above. Mosquito MGs were dissected in PBS 10 to 12 days after mosquito feeding. Next, MGs were incubated with 1% (v/v) of NP40 solution in PBS for 20 min at RT, followed by staining with 1% (v/v) of mercurochrome solution in PBS for 30 min. MGs were then washed and transferred onto glass slides, covered with glass coverslips, and analysed by light microscopy with green filter.

### 4.7 Assessment of in vivo Plasmodium infection

For assessment of in vivo *Plasmodium* hepatic infection, C57BL/6 mice were infected by intravenous injection of 1 × 10^4^ *Pb*WT or *Pb*EXP1ΔCT spz, freshly isolated from the SGs of infected female *A. stephensi* mosquitoes. At 42 hpi, mice were euthanised, and livers were perfused and collected for RNA extraction. For assessment of blood stage infection, C57BL/6 mice were injected i.v. with 1 × 10^4^ *Pb*WT or *Pb*EXP1ΔCT spz or with 1 × 10^3^ or 1 × 10^6^ iRBCs. Parasitaemia was monitored daily by microscopic analysis of Giemsa‐stained blood smears. Mice were also monitored daily for signs of ECM and were euthanised in case of severe disease.

### 4.8 Assessment of gene expression by qRT‐PCR

Collected livers were transferred to RLT buffer supplemented with β‐mercaptoethanol and homogenised using TissueRuptor from QIAGEN. RNA was extracted from liver homogenates by using the RNeasy kit from QIAGEN, according to the manufacturer's instructions. cDNA was synthesized by reverse transcription using the first‐strand cDNA synthesis kit from Thermo Fisher Scientific and employing the following thermocycling parameters: 25°C for 5 min, 37°C for 60 min, and 70°C for 5 min. Liver *P. berghei* load was quantified by qRT‐PCR, employing primers specific for *Pb* 18S rRNA (Table S1). Gene expression levels were normalised to the endogenous mouse housekeeping gene glyceraldehyde‐3‐phosphate dehydrogenase (Table S1) by using the ΔΔC_T_ method. The qRT‐PCR reaction was performed using the SYBR® Green PCR master mix from Applied Biosystems, employing the ABI 7500 Real‐Time PCR System from Life Technologies and qTOWER^3^—Real‐Time PCR Thermal Cycler equipment from Analytik Jena AG, using the following thermocycling parameters: 95°C for 15 min, followed by 40 cycles at 95°C for 15 s, 40 cycles at 55°C for 15 s, 40 cycles at 60°C for 45 s, and 35 cycles at ≥60°C for the melting curve.

### 4.9 Immunofluorescence microscopy

For immunofluorescence microscopy analyses, 1 day prior to infection, 2.5 × 10^4^ or 5 × 10^4^ Huh7 cells were seeded in eight‐well Lab‐Tek chamber slides or on glass coverslips in 24‐well plates, respectively. Cells were infected with 1 × 10^4^ or 2 × 10^4^ spz, respectively, and incubated at 37°C with 5% CO_2_. At 24 and 48 hpi, cells grown in eight‐well Lab‐Tek chamber slides were fixed and permeabilised with ice‐cold methanol for 20 min at RT, washed with 1% (v/v) FCS solution in PBS (washing buffer), and blocked with 10% (v/v) FCS solution in PBS (blocking buffer) for 30 min at 37°C or overnight at 4°C. Primary antibody, mouse anti‐*Pb*HSP70, hybridoma supernatant (1:100, Tsuji et al., 1994), was diluted in blocking buffer and incubated for 1 h at 37°C. Samples were then washed three times with washing buffer and incubated with secondary antibody, goat anti‐mouse Alexa 488 (1:300, Life Technologies, A11029) diluted in blocking buffer, for 1 h at 37°C. Nuclei were stained for 5 min with a Hoechst solution. Finally, cells were washed four times with washing buffer and mounted with 30% (v/v) glycerol solution in PBS. At 30 hpi, cells grown on glass coverslips were fixed with 4% (v/v) paraformaldehyde for 15 min at RT, washed with PBS, and permeabilised and blocked with 0.2% (v/v) Triton X‐100 and 1% (v/v) BSA in PBS (perm/block buffer) for 1 h at RT. Primary antibodies, chicken anti‐*Pb*EXP1 antibody (1:700; provided by Volker Heussler, Institute of Cell Biology, University of Bern, Bern, Switzerland), rabbit monoclonal anti‐ApoH antibody (1:10; Sigma, HPA003732), and goat anti‐UIS4 antibody (1:1,000), were diluted in perm/block buffer and incubated overnight at 4°C. Following three washes with PBS, secondary antibodies were diluted in perm/block buffer and incubated for 40 min at RT. After washing with PBS, coverslips were mounted with Fluoromount G mounting medium. Images were acquired on an Axiovert 200 M widefield fluorescence microscope (Zeiss) or on an LSM 710 confocal laser point‐scanning microscope (Zeiss).

### 4.10 Analysis of UIS4‐EXP1 colocalization and ApoH accumulation

Confocal microscopy images were obtained by sequential scanning of each channel in order to eliminate chromophore crosstalk. Prior to analysis, the background was corrected by applying a threshold value to all channels. UIS4‐EXP1 colocalizing pixels were identified using the “colocalization highlighter” command in ImageJ software and were represented as a percentage of the total EXP1 signal. ApoH accumulation was quantified using the ImageJ software, as previously described (Sa et al., 2017).

### 4.11 Quantification of in vitro merosome formation

For the *in vitro* assessment of merosome formation, Huh7 cells (2.5 × 10^4^) were seeded in eight‐well Lab‐Tek chamber slides, infected with 1 × 10^4^ spz, and incubated at 37°C with 5% CO_2_. At 72 hpi, supernatants were collected and centrifuged at 1,200 rpm for 5 min at RT. Merosomes were then carefully resuspended and counted using a hemocytometer.

### 4.12 Statistical analyses

Statistical significance was evaluated by employing a two‐tailed unpaired *t* test. Values of *P* < .05 were deemed statistically significant. All statistical analyses were performed by using GraphPad Prism 7 software.

## CONFLICT OF INTEREST

The authors declare no competing interests.

## Supporting information

Figure S1. Generation and genotyping of transgenic parasite lines. (a) Schematic representation of the genetic strategy for the attempted generation of the transgenic *Pb*EXP1ΔNT line. Primer binding sites used for genotyping are indicated by thin arrows. (b) Genotype analysis by diagnostic PCR shows mixed populations of PbWT and 5’ and 3’ integrated parasites. B1, gDNA of *Pb*EXP1ΔNT clonal transgenic parasite line; V, vector; WT, gDNA of *Pb*WT parasite line; H_2_O, water control. (c) Schematic representation of the genetic strategy for the generation of the transgenic *Pb*EXP1ΔCT line. Primer binding sites used for genotyping are indicated by thin arrows. CT designates the portion of the gene encoding the C‐terminal domain of the *Pb*EXP1 protein, and C1 and C2 indicate its C1 and C2 regions. dhfr/ts designates the selectable marker. (d) Genotype analysis by diagnostic PCR of the cloned parasite *Pb*EXP1ΔCT line confirms correct 5’ and 3’ integration. B2 and C3, gDNA of the *Pb*EXP1ΔCT clonal transgenic parasite lines; V, vector; WT, gDNA of the *Pb*ANKA parasite line; H_2_O, water control. (e) Successful truncation of the C‐terminal domain of *Pb*EXP1 is shown by the reduction in protein size, using an antibody that was raised against full‐length *Pb*EXP1 (FL – full length). (f) Confirmation of successful truncation in the generated *Pb*EXP1ΔCT parasite line by using an antibody that recognizes exclusively the C‐terminal domain of *Pb*EXP1 (CT). (g) Schematic representation of the process of generation of the transgenic *Pb*_GFP_EXP1ΔCT parasite line. Primer binding sites used for genotyping are indicated by thin arrows. (h) Genotype analysis by diagnostic PCR shows successful 5’ and 3’ integration. C4, gDNA of *Pb*_GFP_EXP1ΔCT clonal transgenic parasite line; V, vector; WT, gDNA of *Pb*_GFP_ parasite line; H_2_O, water control.
Figure S2. Parasitemia and ECM following injection of *Pb*WT‐ and *Pb*EXP1ΔCT‐iRBCs. (a), (b) Parasitemia curves of *Pb*WT and *Pb*EXP1ΔCT parasites following intravenous injection of (a) 1 x 10^3^ and (b) 1 x 10^6^ infected red blood cells (iRBCs) into C57BL/6 mice (n = 3). The percentage of iRBCs was quantified by microscopy analysis of Giemsa‐stained blood smears. (c) Survival rates of C57BL/6 mice following intravenous injection of 1 x 10^3^ or 1 x 10^6^ *Pb*WT‐ and *Pb*EXP1ΔCT‐iRBCs. All mice were age matched.
Figure S3. Impact of CT truncation on *Pb*_GFP_EXP1ΔCT parasite's mosquito and in vitro and in vivo hepatic infection. (a) Number of salivary gland (SG) sporozoites (spz) present in *Pb*GFP and *Pb*_GFP_EXP1ΔCT‐infected mosquitoes. Each symbol represents the average number of SG spz per mosquito from 3 independent mosquito infections, with 36 – 100 mosquitoes dissected per infection. (b) *Pb*_GFP_ and *Pb*_GFP_EXP1ΔCT spz migration from the midgut (MG) to SG. Migration is expressed in percentage and represents the ratio of the number of MG spz 14 days after the blood meal and the number of SG spz 17 days after the blood meal, multiplied by 100. Each symbol represents an independent mosquito infection (n = 3). (c) Number of EEFs in infected Huh7 cells. Quantification was performed at 24 and 48 hpi in four independent wells of eight‐well Lab‐Tek chamber slides. (d) Measurement of EEF size in infected Huh7 cells by immunofluorescence microscopy, at 24 and 48 hpi. Each symbol represents one parasite. n = 40 (for *Pb*_GFP_), 43 (for *Pb*_GFP_EXP1ΔCT) for 24 hpi and n = 41 (for *Pb*_GFP_), 35 (for *Pb*_GFP_EXP1ΔCT) for 48 hpi. (e) Relative parasite burden in the livers of C57BL/6 mice infected with*Pb*_GFP_ and *Pb*_GFP_EXP1ΔCT clonal lines, measured by qRT‐PCR, 42 h after intravenous injection of sporozoites. Parasite burden is represented by the level of transcription of parasite 18S rRNA normalized to *Pb*_GFP_ control, which was set to 100 %. Results are from two independent experiments. Each symbol represents one mouse (n = 3). (a), (b), (c), (d) and (e) were analysed using Two‐tailed unpaired t test. For (a) *P = 0.0471; for (c) left ***P = 0.0007 and right ***P = 0.0003; for (d) **P = 0.0019 and ****P < 0.0001; for (e) ***P = 0.0007; (b) was not significant. Data are shown as mean ± SEM.
Figure S4. Impact of CT truncation on the motility of *Pb*EXP1ΔCT sporozoites in the mosquito. *Pb*WT and *Pb*EXP1ΔCT spz migration from the midgut (MG) to the salivary glands (SG). Migration is expressed in percentage and represents the ratio of the number of MG spz 14 days after the blood meal and the number of SG spz 17 days after the blood meal, multiplied by 100. Each symbol represents an independent mosquito infection (n = 4). Results were analysed using Two‐tailed unpaired t test. n.s., not significant. Data are shown as mean ± SEM.
Figure S5. Impact of CT truncation on the internalization of Apolipoprotein H (ApoH) by *Pb*EXP1ΔCT liver stages. ApoH internalization was assessed by immunofluorescence microscopy at 30 hpi, in Huh7 cells infected with 2 x 10^4^ sporozoites of *Pb*WT, *Pb*EXP1ΔC2, and *Pb*EXP1ΔCT parasite lines. The internalized ApoH was calculated as the difference between the total ApoH signal inside the exoerythrocytic form (EEF) and the ApoH signal in an area of the same size and shape outside the EEF and normalized to the EEF area. Results shown are from two coverslips infected in one experiment. Each symbol represents one parasite (n = 25). Results were analysed using Two‐tailed unpaired t test. n.s., not significant; ****P < 0.0001. Data are shown as mean ± SEM.
Table S1. List of primers.
