# Supplementary material for: A crucial role for the C‐terminal domain of exported protein 1 during the mosquito and hepatic stages of the Plasmodium berghei life cycle
Source: Cell Microbiol. 2019 Jul 30;21(10):e13088. doi: 10.1111/cmi.13088 (PMC6771729; doi:10.1111/cmi.13088)
Supplement: Supplementary file 1 — Figure S1. Generation and genotyping of transgenic parasite lines. (a) Schematic representation of the genetic strategy for the attempted generation of the transgenic PbEXP1ΔNT line. Primer binding sites used for genotyping are indicated by thin arrows. (b) Genotype analysis by diagnostic PCR shows mixed populations of PbWT and 5’ and 3’ integrated parasites. B1, gDNA of PbEXP1ΔNT clonal transgenic parasite line; V, vector; WT, gDNA of PbWT parasite line; H2O, water control. (c) Schematic representation of the genetic strategy for the generation of the transgenic PbEXP1ΔCT line. Primer binding sites used for genotyping are indicated by thin arrows. CT designates the portion of the gene encoding the C‐terminal domain of the PbEXP1 protein, and C1 and C2 indicate its C1 and C2 regions. dhfr/ts designates the selectable marker. (d) Genotype analysis by diagnostic PCR of the cloned parasite PbEXP1ΔCT line confirms correct 5’ and 3’ integration. B2 and C3, gDNA of the PbEXP1ΔCT clonal transgenic parasite lines; V, vector; WT, gDNA of the PbANKA parasite line; H2O, water control. (e) Successful truncation of the C‐terminal domain of PbEXP1 is shown by the reduction in protein size, using an antibody that was raised against full‐length PbEXP1 (FL – full length). (f) Confirmation of successful truncation in the generated PbEXP1ΔCT parasite line by using an antibody that recognizes exclusively the C‐terminal domain of PbEXP1 (CT). (g) Schematic representation of the process of generation of the transgenic Pb GFPEXP1ΔCT parasite line. Primer binding sites used for genotyping are indicated by thin arrows. (h) Genotype analysis by diagnostic PCR shows successful 5’ and 3’ integration. C4, gDNA of Pb GFPEXP1ΔCT clonal transgenic parasite line; V, vector; WT, gDNA of Pb GFP parasite line; H2O, water control. Figure S2. Parasitemia and ECM following injection of PbWT‐ and PbEXP1ΔCT‐iRBCs. (a), (b) Parasitemia curves of PbWT and PbEXP1ΔCT parasites following intravenous injection o [file CMI-21-na-s001.docx]

**Supplementary Material**

**A crucial role for the C-terminal domain of EXP1 during the mosquito and hepatic stages of the *Plasmodium berghei* life cycle**

Kamil Wolanin, Diana Fontinha, Margarida Sanches-Vaz, Britta Nyboer, Kirsten Heiss,

Ann-Kristin Mueller, Miguel Prudêncio


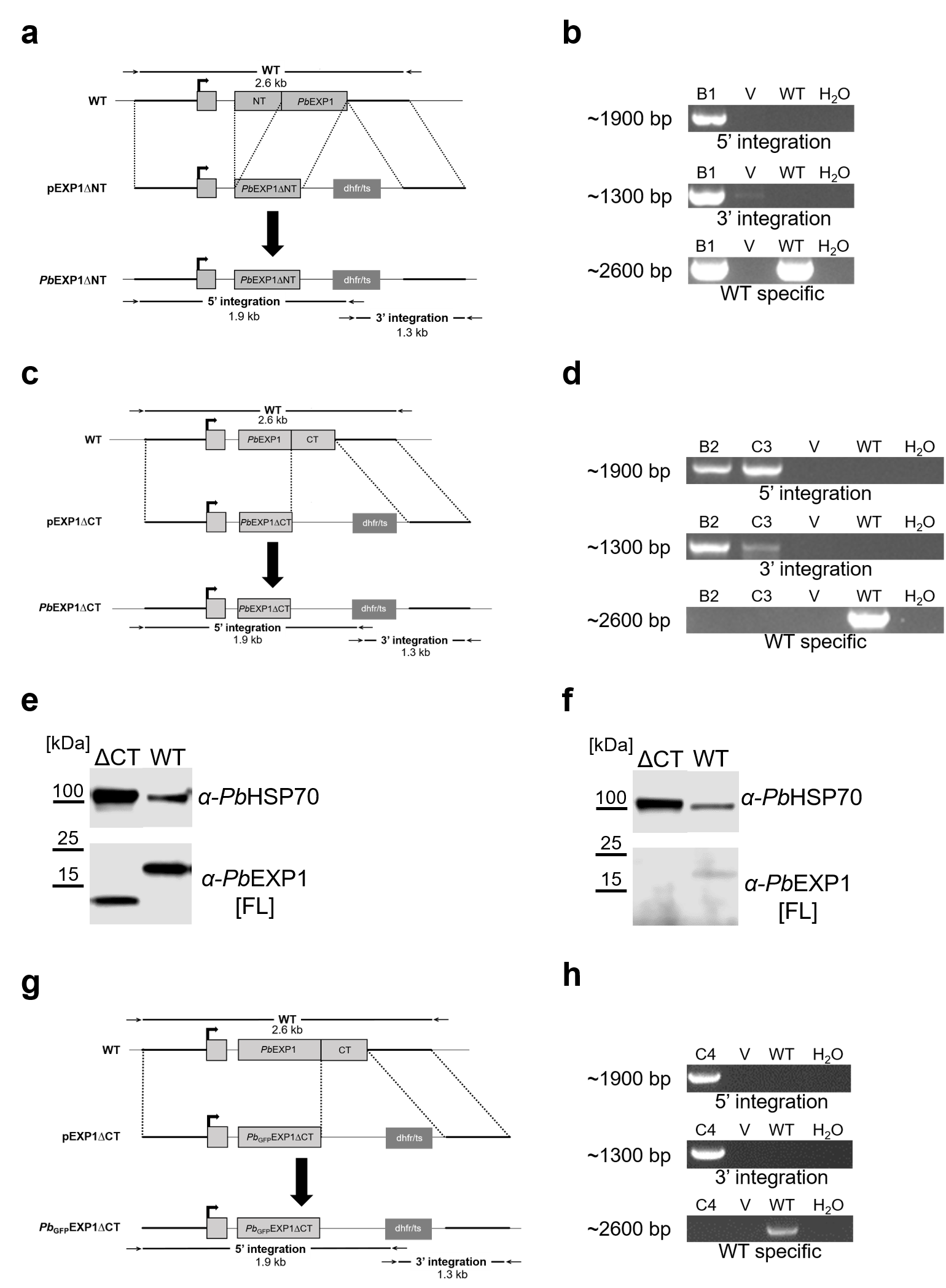


**FIGURE S1. Generation and genotyping of transgenic parasite lines**. **(a)** Schematic representation of the genetic strategy for the attempted generation of the transgenic *Pb*EXP1ΔNT line. Primer binding sites used for genotyping are indicated by thin arrows. **(b)** Genotype analysis by diagnostic PCR shows mixed populations of *Pb*WT and 5’ and 3’ integrated parasites. B1, gDNA of *Pb*EXP1ΔNT clonal transgenic parasite line; V, vector; WT, gDNA of *Pb*WT parasite line; H_2_O, water control. **(c)** Schematic representation of the genetic strategy for the generation of the transgenic *Pb*EXP1ΔCT line. Primer binding sites used for genotyping are indicated by thin arrows. CT designates the portion of the gene encoding the C-terminal domain of the *Pb*EXP1 protein, and C1 and C2 indicate its C1 and C2 regions. *dhfr/ts* designates the selectable marker. **(d)** Genotype analysis by diagnostic PCR of the cloned parasite *Pb*EXP1ΔCT line confirms correct 5’ and 3’ integration. B2 and C3, gDNA of the *Pb*EXP1ΔCT clonal transgenic parasite lines; V, vector; WT, gDNA of the *Pb*ANKA parasite line; H_2_O, water control. **(e)** Successful truncation of the C-terminal domain of *Pb*EXP1 is shown by the reduction in protein size, using an antibody that was raised against full-length *Pb*EXP1 (FL – full length). **(f)** Confirmation of successful truncation in the generated *Pb*EXP1ΔCT parasite line by using an antibody that recognizes exclusively the C-terminal domain of *Pb*EXP1 (CT). **(g)** Schematic representation of the process of generation of the transgenic *Pb*_GFP_EXP1ΔCT parasite line. Primer binding sites used for genotyping are indicated by thin arrows. **(h)** Genotype analysis by diagnostic PCR shows successful 5’ and 3’ integration. C4, gDNA of *Pb*_GFP_EXP1ΔCT clonal transgenic parasite line; V, vector; WT, gDNA of *Pb*_GFP_ parasite line; H_2_O, water control.


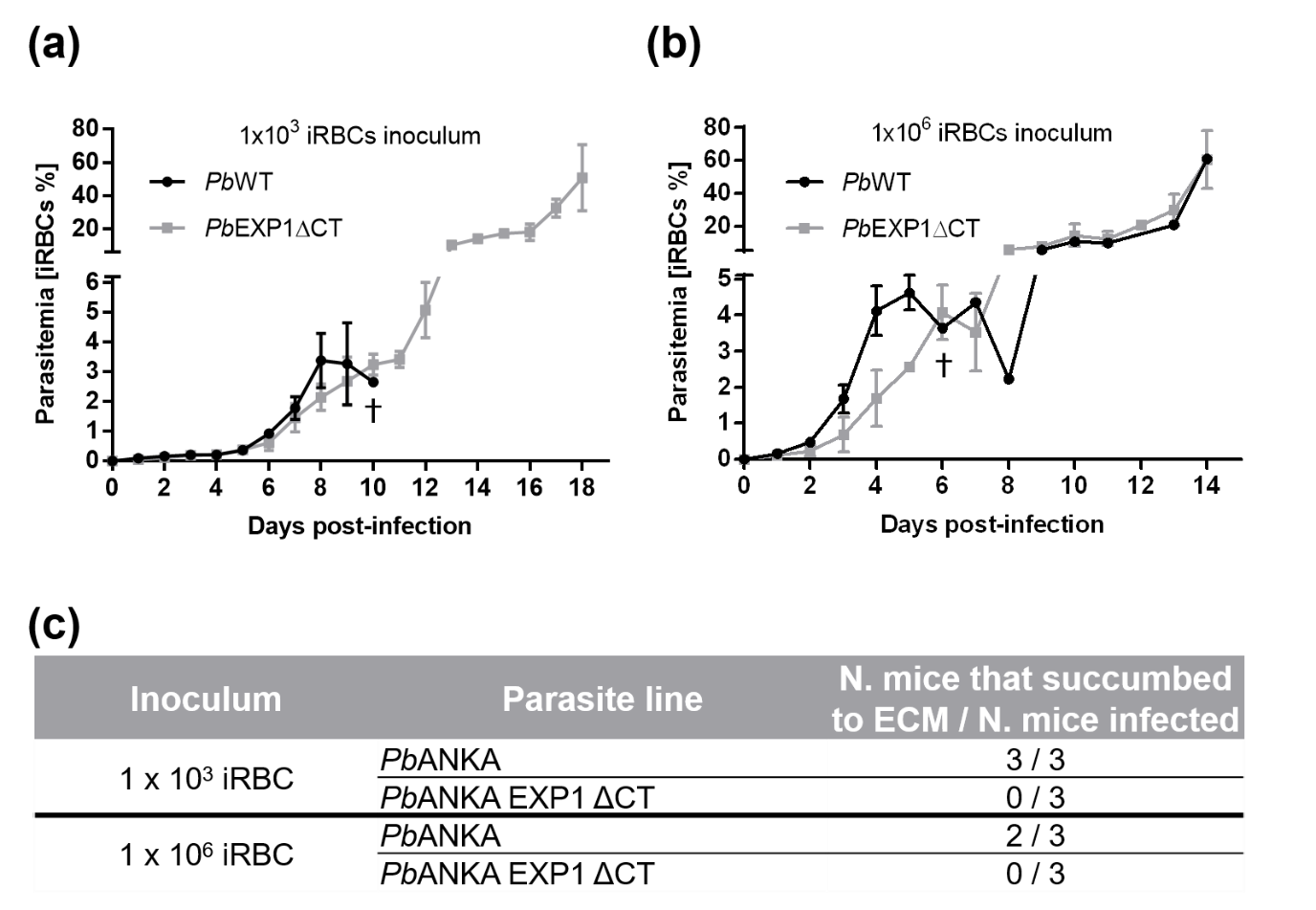


**FIGURE S2. Parasitemia and ECM following injection of *Pb*WT- and *Pb*EXP1ΔCT-iRBCs.** **(a)**, **(b)** Parasitemia curves of *Pb*WT and *Pb*EXP1ΔCT parasites following intravenous injection of **(a)** 1 x 10^3^ and **(b)** 1 x 10^6^ infected red blood cells (iRBCs) into C57BL/6 mice (n = 3). The percentage of iRBCs was quantified by microscopy analysis of Giemsa-stained blood smears. **(c)** Survival rates of C57BL/6 mice following intravenous injection of 1 x 10^3^ or 1 x 10^6^ *Pb*WT- and *Pb*EXP1ΔCT-iRBCs. All mice were age matched.


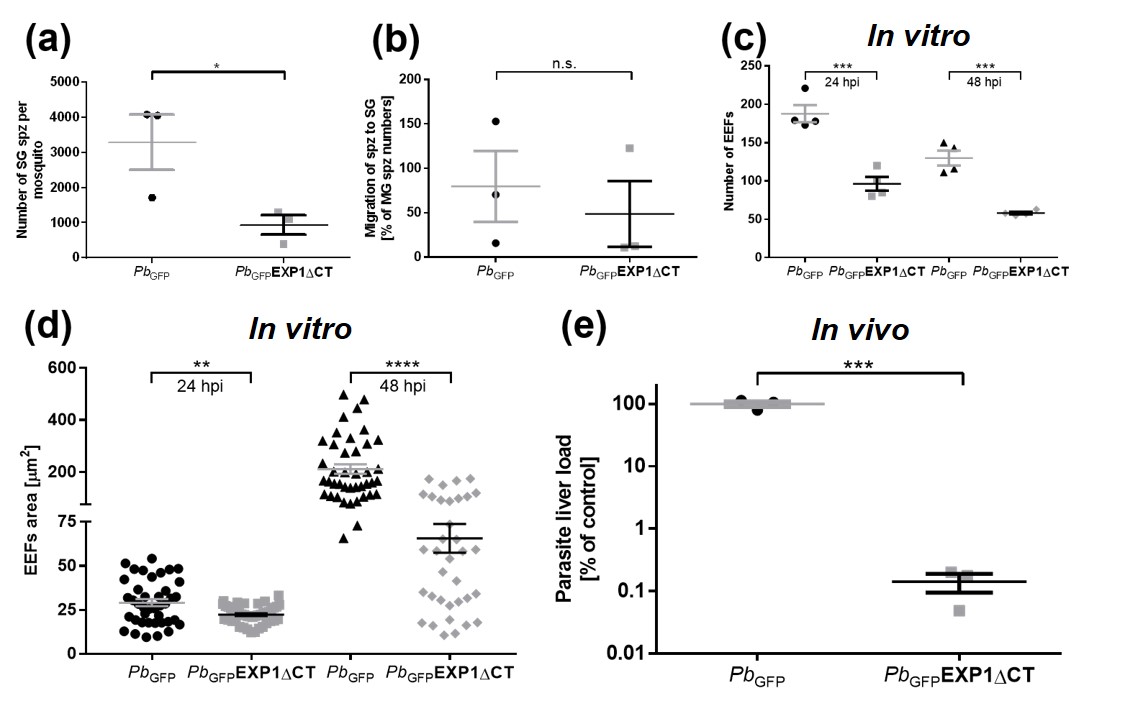


**FIGURE S3. Impact of CT truncation on *Pb*_GFP_EXP1ΔCT parasite’s mosquito and *in vitro* and *in vivo* hepatic infection**. **(a)** Number of salivary gland (SG) sporozoites (spz) present in *Pb*_GFP_ and *Pb*_GFP_EXP1ΔCT-infected mosquitoes. Each symbol represents the average number of SG spz per mosquito from 3 independent mosquito infections, with 36 – 100 mosquitoes dissected per infection. **(b)** *Pb*_GFP_ and *Pb*_GFP_EXP1ΔCT spz migration from the midgut (MG) to SG. Migration is expressed in percentage and represents the ratio of the number of MG spz 14 days after the blood meal and the number of SG spz 17 days after the blood meal, multiplied by 100. Each symbol represents an independent mosquito infection (n = 3). **(c)** Number of EEFs in infected Huh7 cells. Quantification was performed at 24 and 48 hpi in four independent wells of eight-well Lab-Tek chamber slides. **(d)** Measurement of EEF size in infected Huh7 cells by immunofluorescence microscopy, at 24 and 48 hpi. Each symbol represents one parasite. n = 40 (for *Pb*_GFP_), 43 (for *Pb*_GFP_EXP1ΔCT) for 24 hpi and n = 41 (for *Pb*_GFP_), 35 (for *Pb*_GFP_EXP1ΔCT) for 48 hpi. **(e)** Relative parasite burden in the livers of C57BL/6 mice infected with *Pb*_GFP_ and *Pb*_GFP_EXP1ΔCT clonal lines, measured by qRT-PCR, 42 h after intravenous injection of sporozoites. Parasite burden is represented by the level of transcription of parasite 18S rRNA normalized to *Pb*_GFP_ control, which was set to 100 %. Results are from two independent experiments. Each symbol represents one mouse (n = 3). **(a)**, **(b)**, **(c)**, **(d)** and **(e)** were analysed using Two-tailed unpaired t test. For **(a)** **P* = 0.0471; for **(c)** left ****P* = 0.0007 and right ****P* = 0.0003; for **(d)** ***P* = 0.0019 and *****P* < 0.0001; for **(e)** ****P* = 0.0007; **(b)** was not significant. Data are shown as mean ± SEM.


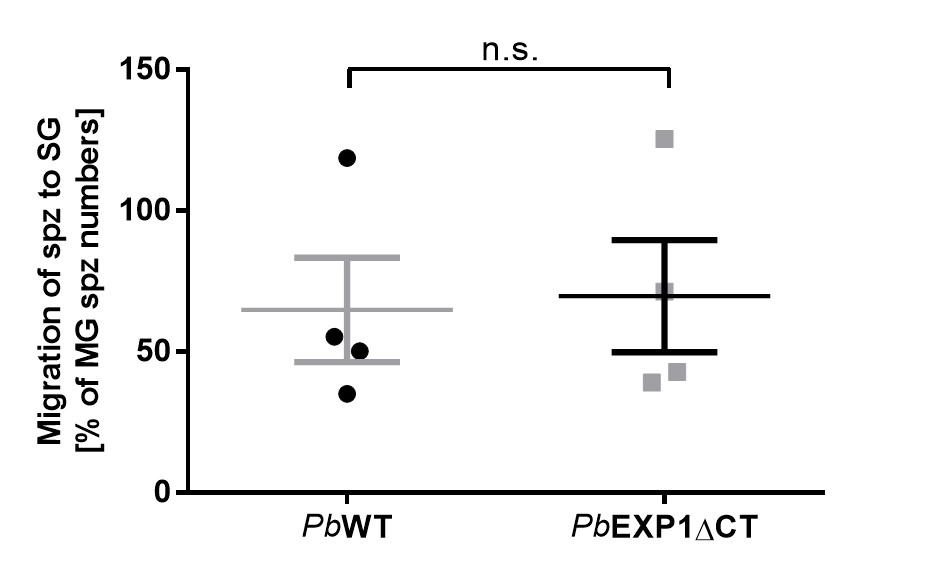


**FIGURE S4. Impact of CT truncation on the motility of *Pb*EXP1ΔCT sporozoites in the mosquito**. *Pb*WT and *Pb*EXP1ΔCT spz migration from the midgut (MG) to the salivary glands (SG). Migration is expressed in percentage and represents the ratio of the number of MG spz 14 days after the blood meal and the number of SG spz 17 days after the blood meal, multiplied by 100. Each symbol represents an independent mosquito infection (n = 4). Results were analysed using Two-tailed unpaired t test. n.s., not significant. Data are shown as mean ± SEM.


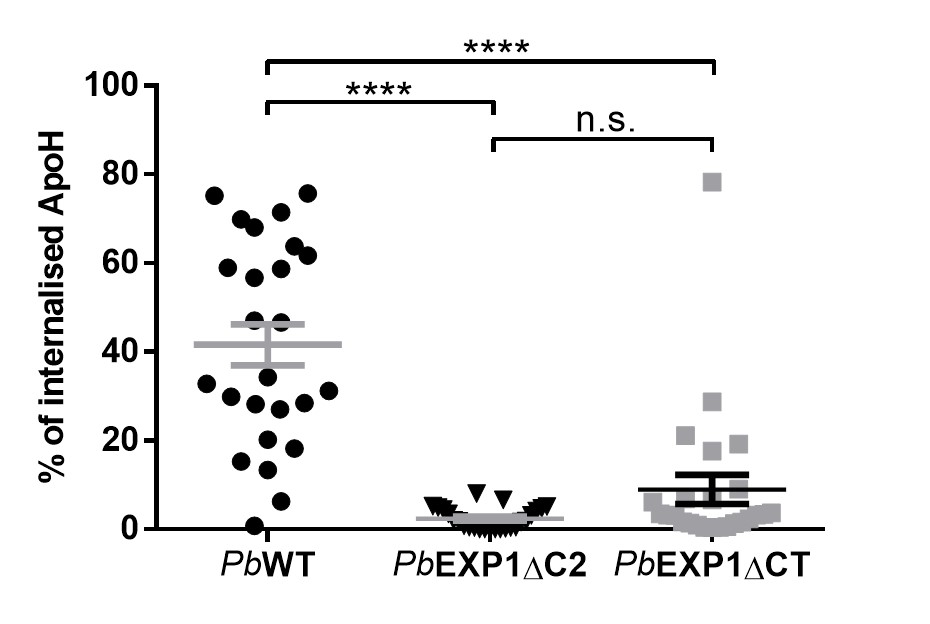


**FIGURE S5. Impact of CT truncation on the internalization of Apolipoprotein H (ApoH) by *Pb*EXP1ΔCT liver stages.** ApoH internalization was assessed by immunofluorescence microscopy at 30 hpi, in Huh7 cells infected with 2 x 10^4^ sporozoites of *Pb*WT, *Pb*EXP1ΔC2, and *Pb*EXP1ΔCT parasite lines. The internalized ApoH was calculated as the difference between the total ApoH signal inside the exoerythrocytic form (EEF) and the ApoH signal in an area of the same size and shape outside the EEF and normalized to the EEF area. Results shown are from two coverslips infected in one experiment. Each symbol represents one parasite (n = 25). Results were analysed using Two-tailed unpaired t test. n.s., not significant; *****P* < 0.0001. Data are shown as mean ± SEM.

**TABLE S1.** List of primers.

| Experiment | Gene (gene no./ID) | Primer name | Sequence |
| --- | --- | --- | --- |
| pEXP1ΔCT plasmid | *Pb*EXP1 (PBANKA_092670) | 3’UTR *Pb*EXP1for | **5’**-CCATCGATGTATCATAAAAAGTTTCGACTC-**3’** |
|  | *Pb*EXP1 (PBANKA_092670) | 3’UTR *Pb*EXP1rev | **5’**- GGGGTACCGAGTAAATACCCCTACATATG-**3’** |
|  | *Pb*EXP1 (PBANKA_092670) | 5’UTR *Pb*EXP1 ORF dC2for | **5’**-ATAAGAATGCGGCCGCGCCGTTTAACTCTTAATTTAC-**3’** |
|  | *Pb*EXP1 (PBANKA_092670) | 5’UTR *Pb*EXP1 ORF dCTrev | **5’**-CGGGATCCTTATCTAGAGTACATAACTAATCCAGCACC-**3’** |
| pEXP1ΔNT plasmid | *Pb*EXP1 (PBANKA_092670) | 3’UTR *Pb*EXP1for | **5’**-CCATCGATGTATCATAAAAAGTTTCGACTC-**3’** |
|  | *Pb*EXP1 (PBANKA_092670) | 3’UTR *Pb*EXP1rev | **5’**-GGGGTACCGAGTAAATACCCCTACATATG-**3’** |
|  | *Pb*EXP1 (PBANKA_092670) | 5’UTR SP *Pb*EXP1for | **5’**-ATAAGAATGCGGCCGCCCTATGCATGCATCTATTGTTC-**3’** |
|  | *Pb*EXP1 (PBANKA_092670) | 5’UTR SP *Pb*EXP1rev | **5’**-GGACTAGTAACATTTTTGGAGCCAGTTTTACC-**3’** |
|  | *Pb*EXP1 (PBANKA_092670) | TM CT *Pb*EXP1for | **5’**-GGACTAGTGTAGCCCTTGCCACAGC-**3’** |
|  | *Pb*EXP1 (PBANKA_092670) | TM CT *Pb*EXP1rev | **5’**-CGGGATCCTCATTGTTGAAGATTTGGCATG-**3’** |
| Genotyping ΔCT/ΔNT parasite lines | 5’ integration | *Pb*EXP-1 int testfor | **5**′-GCTCGCTCTTTAGAATTTCAC-**3**′ |
|  | 5’ integration | b3D^+^rev | **5**′-CCTTGCTCATTTACCTGCTAATACGATTGC-**3**′ |
|  | 3’ integration | Tgfor | **5**′-CGCATTATATGAGTTCATTTTACACAATCC-**3**′ |
|  | 3’ integration | *Pb*EXP-1 int testrev | **5**′-GCTTATATCGATTTGTGCTAACTGG-**3**′ |
|  | WT | *Pb*EXP-1 int testfor | **5**′-GCTCGCTCTTTAGAATTTCAC-**3**′ |
|  | WT | *Pb*EXP-1 int testrev | **5**′-GCTTATATCGATTTGTGCTAACTGG-**3**′ |
| qRT-PCR | MmGAPDH (14433) | *Mm*GAPDHfor | **5**′-CGTCCCGTAGACAAAATGGT-**3**′ |
|  | MmGAPDH (14433) | *Mm*GAPDHrev | **5**′-TTGATGGCAACAATCTCCAC-**3**′ |
|  | 18S rRNA | *Pb*18SrRNAfor | **5**′-AAGCATTAAATAAAGCGAATACATCCTTAC-**3**′ |
|  | 18S rRNA | *Pb*18SrRNArev | **5**′-GGAGATTGGTTTTGACGTTTATGTG-**3**′ |
